# Significance of quality of care for quality of life in persons with dementia at risk of nursing home admission: a cross-sectional study

**DOI:** 10.1186/s12912-017-0230-6

**Published:** 2017-07-14

**Authors:** Christina Bökberg, Gerd Ahlström, Staffan Karlsson

**Affiliations:** 1 Department of Health Sciences, Faculty of Medicine, Lund University, PO Box 157, -221 00 Lund, SE Sweden; 2 School of Health and Welfare, Halmstad University, PO Box 823, -301 18 Halmstad, SE Sweden

**Keywords:** Quality of life, Quality of care, Persons with dementia, Home care

## Abstract

**Background:**

Quality of life in persons with dementia is, in large part, dependent on the quality of care they receive. Investigating both subjective and objective aspects of quality of care may reveal areas for improvement regarding their care, which information may ultimately enable persons with dementia to remain living in their own homes while maintaining quality of life. The aim of this study was to 1) describe self-reported quality of life in persons with dementia at risk of nursing home admission. 2) describe subjective and objective aspects of quality of care, 3) investigate the significance of quality of care for quality of life.

**Methods:**

A cross-sectional interview study design was used, based on questionnaires about quality of life (QoL-AD) and different aspects of quality of care (CLINT and quality indicators). The sample consisted of 177 persons with dementia living in urban and rural areas in Skåne County, Sweden. Descriptive and comparative statistics (Mann-Whitney U-test) were used to analyse the data.

**Results:**

Based upon Lawton’s conceptual framework for QoL in older people, persons with pain showed significantly lower quality of life in the dimensions behavioural competence (*p* = 0.026) and psychological wellbeing (*p* = 0.006) compared with those without pain. Satisfaction with care seemed to have a positive effect on quality of life. The overall quality of life was perceived high even though one-third of the persons with dementia had daily pain and had had a weight loss of ≥4% during the preceding year. Furthermore, 23% of the persons with dementia had fallen during the last month and 40% of them had sustained an injury when falling.

**Conclusion:**

This study indicates need for improvements in home care and services for persons with dementia at risk for nursing home admission. Registered nurses are responsible for nursing interventions related to pain, patient safety, skin care, prevention of accidents, and malnutrition. Therefore, it is of great importance for nurses to have knowledge about areas that can be improved to be able to tailor interventions and thereby improve quality of care outcomes such as quality of life in persons with dementia living at home.

## Background

Persons with dementia, at risk of nursing home admission need health care and social services of highest quality to maintain or, better, improve their quality of life (QoL) [1, 2]. Their need for security in the care experience as well as support for the informal caregiver should govern the design of health care. There is a need to further explore QoL in home care settings, since previous research has tended to focus more on QoL in nursing home environments [3–5]. Dementia is strongly related to old age and a serious chronic condition affecting all aspects of daily living [6]. Because of deterioration in cognition, function and behaviour, persons with dementia have complex needs for health care and social services. Compared with older persons without dementia they need more personal care, more hours of care and more supervision, all of which requirements are associated with greater caregiver strain [7]. The need for help with activities of daily living (ADLs) starts early in the disease course and evolves constantly over time [6]. Receiving help with ADLs from others has been found to be significantly related to low QoL, as has not being able to remain alone at home without help [8].

Neuropsychiatric symptoms, cognitive impairment and dependency have been found to predict the risk of institutionalization in persons with dementia. Moreover, informal caregiver experiences of burden and/or strain seem to predict the care recipient moving into institutional care. Furthermore, when formal health care and social services are insufficient and fail to meet the person with dementia’s needs, the risk of nursing home admission appears to increase [9, 10]. At present, home care is put forward as the best way of caring for persons with dementia, based on both providing a better QoL and for being more cost-effective compared with institutional care [11, 12]. Still, research is contradictory regarding the person’s QoL when remaining at home rather than moving into a nursing home since the reasons for nursing home admission differ [9, 10]. Some older people prefer home care instead of any other option, since home is a place of emotional and physical associations, memories, and comfort. Although, when older people realize that a nursing home is a better option, leaving home can be disruptive and depressing [12].

To understand QoL in old age, not only the distress and impairments resulting from poor health, but also non-health-related aspects need to be considered [13]. Quality of life is commonly viewed and assessed as a multidimensional concept [14–17] encompassing different domains (emotional, physical, social, and environmental) of a person’s wellbeing [17]. Today these are considered crucial outcome measures for health service research. This reflects concerns about capturing important ways in which health care conditions impact on a person’s QoL and can bring about meaningful understanding to change treatments [18].

**Fig. 1:**
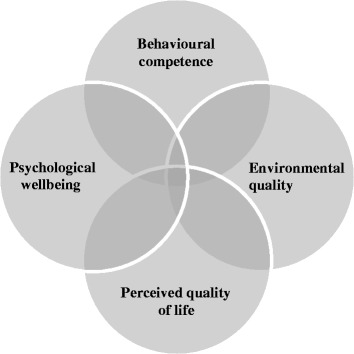
Dimensions of Quality of life in persons with dementia according to Lawton’s model about here

Lawton [16, 17] describes a conceptual framework for QoL in older people, including four domains of importance (Fig. 1). The first domain is *behavioural competence*: how well a person functions in the domains of physical health, ADLs, cognition, and social behaviour. The second domain is *environmental quality*, which includes housing quality. The third domain is *perceived quality of life* and entails the evaluation of one’s neighbourhood, family, friends, etc. The fourth domain is *psychological wellbeing*: the global aspects of mental health. Each of these domains is highly relevant to evaluating QoL in persons with dementia.

The QoL in vulnerable older people, such as persons with dementia, may be improved by high quality of care (QoC), among others [1, 2]. Quality of care can be defined as the degree to which health care and social services for individuals and populations increase the likelihood of desired health outcomes and are consistent with current professional knowledge [1]. Quality of care indicators are objective measures that reflect care standards and are used as guides to monitor and evaluate the QoC [19, 20]. These indicators show how structure and processes impact on a person’s wellbeing, health and/or QoL. Quality of care indicators can also bring about meaningful understanding that can lead to changes in treatment [18]. Important QoC indicators in the care of older people are pain, falls, pressure ulcers and weight loss, indicating deterioration in chronic conditions such as dementia [21–26].

Another way of measuring QoC is satisfaction with care. There is no universally accepted definition of or measure for satisfaction with care, but a care recipient’s satisfaction is none the less regarded as an important aspect of QoC. While some researchers focus on care recipients’ satisfaction with the quality and type of health care services received, others focus on people’s satisfaction with the health system more generally [27]. In this study, satisfaction with health care and social services is understood to concern care recipients’ and informal caregivers’ experience of utilized care in relation to their expectations and needs [18]. Therefore, by investigating both subjective and objective aspects of QoC we may reveal areas for improvement regarding health care and social services at home. Such information may ultimately enable persons with dementia to remain living in their own homes while maintaining QoL, since QoL in persons with dementia is, in large part, dependent on the QoC they receive [1, 2].

### Aim

This study aims to 1) describe self-reported QoL in persons with dementia at risk of nursing home admission, 2) describe subjective and objective aspects of QoC and 3) investigate the significance of QoC for QoL in persons with dementia at risk of nursing home admission.

## Methods

### Design

A cross-sectional study design was used, based on a structured interview with persons with dementia at risk of nursing home admission, and their informal caregiver as proxy raters.

### Setting

The responsibility for the Swedish welfare system is shared by the central government, county councils (*n* = 20) and municipalities (*n* = 290). The role of the central government is to establish principles and guidelines, and to set the political agenda for health and medical care. Access to formal care and social services are based on assessments of individual needs and being available to all members of society on equal terms [28] The county councils are largely divided into hospital care, out-patient specialist care and primary care and are responsible for health care delivery such as assessments leading to dementia diagnosis, treatment, and follow-up. The municipalities are responsible for providing assistance for those older persons who are receiving formal health care and social services at home, in day care or are living in a nursing home [29]. Most of the formal care providers working in home care in Sweden are assistant nurses [30] providing health care and social services including help with IADLs, PADLs and medical treatments [31]. Other formal care providers are registered nurses in charge of home nursing care (e.g. administering wound dressings, injections), social workers, occupational therapists, and physiotherapists in charge of rehabilitation and needs assessments [32]. The care of persons with dementia is guided by the Swedish National Guidelines for Care in Cases of Dementia [33].

### Participants

Inclusion criteria in this study were persons with dementia ≥65 years old living at home, receiving formal health care and social services. An additional criterion was being at risk of nursing home admission within six months as per the assessment of their formal nursing caregiver being familiar with the person with dementia situation, having a dementia diagnosis, with a Standardized Mini Mental State Examination (S-MMSE) [34, 35] score ≤ 24, and having an informal caregiver visiting at least twice a month. Both persons living in urban and persons in rural areas in Skåne County, Sweden, being cared for by either a public home care organization or private home care entrepreneurs, were included in the sample. An exclusion criterion was Korsakoff’s syndrome.

Of the approached 243 participants, 66 dropped out. In the time between being invited to participate in the study and being contacted by a researcher, twelve persons with dementia had moved into a nursing home and four had deceased. The remaining 50 drop-outs had either changed their minds or were too tired to participate. The drop-outs consisted of 52% women with dementia, the same proportion as for those included in the study. No further information on the drop-outs is available. In total, 177 persons with dementia were included in the study.

### Measurements

#### Background questions

Table 1 presents participants’ socio-demographic background characteristics including age, gender, marital status, living conditions, type of dementia and information about whether the person with dementia was on a waiting list for nursing home placement. To assess cognitive impairment, we used S-MMSE scores [34, 35]**.** The possible score ranges from 0 to 30. Higher scores indicate less cognitive impairment. Functional independence was measured using the Katz Index of Independence in Activities of Daily Living [36]. The possible total sum of this scale ranges from 0 to 6. Higher scores on this scale indicate greater independency in ADL.

**Table 1:** Characteristics of the persons with dementia (*n* = 177)

Background variable		
Age, yrs.	Median	(Q1-Q3)
	82	(78-86)
Gender	N	%
Female	92	(52)
Marital status		
Married	122	(69)
Widowed	45	(25)
Divorced	6	(3)
Unmarried	3	(2)
Unknown	1	(1)
Living conditions		
Living with informal caregiver	120	(68)
Living alone	53	(30)
Other	4	(2)
Type of dementia^a^		
Alzheimer’s disease	78	(46)
Vascular dementia	56	(33)
Alzheimer’s disease + vascular dementia	12	(7)
Fronto-temporal dementia	2	(1)
Lewy body dementia	2	(1)
Unknown	9	(5)
Other	12	(7)
On a waiting list for nursing home admission^b^	24	(14)
S-MMSE score,^d^ range 0–30 ^c^	Median	(Q1-Q3)
Total score	16	(11-20)
KATZ-ADL score, range 0–6 ^c^		
Total score	4	(2-5)

^a^Missing *n* = 6;

^b^missing *n* = 3

^c^The underlined score is the most favourable score

^d^Missing *n* = 10 (unable to complete the Standardized Mini Mental State Examination (S-MMSE) owing to cognitive problems related to dementia

KATZ-ADL = Katz Index of Independence in Activities of Daily Living; Q1 = first quartile; Q3 = third quartile

#### Quality of life (QoL)

Quality of life was assessed by the persons with dementia using the Quality of Life in Alzheimer’s Disease (QoL-AD) scale [37, 38]. The instrument consists of 13 items relating to physical health, energy level, mood, living situation, memory, relationships with spouse, friends, and family, self as a whole, ability to do chores around the house, ability to do things for fun, financial situation, and life as a whole. Each item is measured on a 4-point scale ranging from 1 = poor to 4 = excellent. The total score ranges from 13 to 52, with higher scores indicating a higher QoL.

Drawing upon Lawton’s [15] model of QoL, the 13 items in the QoL-AD were sorted into four categories: *behavioural competence* contained the items physical health, energy level, memory, ability to do chores around the house, and ability to do things for fun. *Environmental quality* consisted of the items living situation and financial situation. *Perceived quality of life* contained the items relationships with spouse, friends, and relationships with family. *Psychological wellbeing* contained the items mood, self as a whole, and life as a whole.

The internal consistency reliability for the QoL-AD was calculated using Cronbach’s alpha. For all 13 items on the scale, α was 0.82, which is in line with previous research on the original 13-item QoL-AD measure (α = 0.88) for persons with Alzheimer’s disease [37]. The results for the four dimensions were: behavioural competence (five items), α = 0.67; environmental quality (two items), α = 0.70; perceived QoL (three items), α = 0.68; and psychological wellbeing (three items), α = 0.59. Values above α = 0.7 are considered acceptable; however, values >0.8 are preferable [39].

#### Quality of care (QoC)

Quality of care was assessed in three ways. Firstly, for the subjective judgement of the informal caregiver, we used an adapted version of the Client Interview (CLINT) instrument [2]. The CLINT for the home care setting consists of nine questions concerning satisfaction of informal caregiver with the health care and social services received by the person with dementia. The questions concern quality of interaction with staff, hygiene, cleaning, gardening, and food; also, there is a general question about satisfaction with care. The response alternatives are “yes, always”, “yes, usually”, “sometimes”, “seldom” and “never”. The total score ranges from 9 to 45. The higher the score, the lower the rated QoC.

The internal consistency reliability for the CLINT for all nine items on the scale was α = 0.59. The item gardening had a high frequency of missing values (*n* = 134) and was therefore removed from our analysis. Cronbach’s alpha after the exclusion was α = 0.70.

Our second way of assessing QoC was by asking one question about dementia-specific care: “Do you or your relative use any dementia-specific care (such as day care or respite care)?” The answer alternatives to this question were “yes” and “no”. The answer “yes” was followed up by one question about satisfaction with received dementia-specific care. Response alternatives were: “very dissatisfied”, “dissatisfied”, “neither satisfied nor dissatisfied”, “satisfied” and “very satisfied”.

As a third way of assessing QoC, we evaluated QoC indicators including presence of pain, fall, pressure ulcer, and weight loss. Pain was evaluated by asking how often the person with dementia had expressed signs of pain in the last seven days. Response alternatives were “no pain”, “no daily pain” and “daily pain”. The question regarding fall was, “Has the person with dementia fallen in the past month?” Response alternatives were “yes” and “no”. “Yes” for fall was followed up with a question to find out if the person had sustained injury when falling, with response alternatives “yes” and “no”. In addition, questions about presence of pressure ulcers and weight loss of ≥4% in the previous year were answered by “yes” or “no”.

### Procedure for the data collection

Data were collected between January 2011 and January 2013. The recruitment of participants was done through 15 contact persons; registered nurses specialized in dementia care, in twelve municipalities. The contact persons asked formal caregivers, i.e. registered nurses, and social workers, who were well known to the person with dementia, to give verbal information about the study to the person with dementia and their informal caregiver. They were also asked if a researcher could contact them to give more detailed information about the study and implications of participation. The formal caregivers gave the information back to the contact persons, who in turn contacted the researchers. After verbal permission, the informal caregiver was contacted by phone by a researcher who gave detailed information about the study and asked for verbal consent for participation; the time and place of the interview was then agreed. Just before the interview the researcher again clarified the purpose of the interview, both verbally and written, and gave the participants opportunity to ask questions before signing the informed consent.

Nine specific trained researchers interviewed the person with dementia and informal caregiver via face-to-face interviews in the person with dementia’s own home or at a day care facility. The researchers asked questions, starting with the person with dementia, answering the questionnaires S-MMSE and QoL-AD. Remaining questionnaires were answered by the informal caregiver as a proxy rater.

### Statistical analyses

Not all questionnaires were filled out or answered completely and several individual items had missing data. When the total score was calculated a maximum of one missing item in the QoL-AD and CLINT instruments was replaced by the mean score of the remaining items of the participant. Where more than one item was missing, no total score for the QoL-AD and CLINT or for any of the individual QoL-AD dimensions was calculated. Since the item gardening was excluded from the CLINT the total score, the total score in this study ranged from 8 to 40.

The QoC indicators fall, injuries from falling, and weight loss of ≥4% were dichotomized into “present” and “not present”. Pain was dichotomized into “no pain” (“no pain” and “no daily pain”) and “daily pain”. The median total score, 14, for the CLINT was used to dichotomize satisfaction with care into two groups, “high satisfaction” (score 0–13) and “low satisfaction” (score 14–40). Responses to dementia-specific care questions were dichotomized into “yes” and “no”.

Since the sample was not normal distributed, the Mann-Whitney U-test was applied to compare the QoC indicators and the perceptions of the significance of care for the four QoL dimensions, as well as for the total QoL-AD score. Only one person had a pressure ulcer and therefore was this indicator excluded from the analysis. A *p*-value of ≤0.05 was considered statistically significant. For data analysis, IBM SPSS Statistics for Windows, version 23.0 (IBM Corp., Armonk, NY, USA), was used.

## Results

The participants consisted of 52% women aged 65–98 years. Most of them were either married or widowed and the most common living condition was living together with the informal caregiver, followed by living alone. Alzheimer’s disease was the most reported dementia diagnosis, followed by vascular dementia. Fourteen per cent of participants were on a waiting list for nursing home placement. The median total KATZ-ADL score was 4; the median S-MMSE score was 16 (Table 1).

The persons with dementia had a total median score of 36 (first quartile (Q1) – third quartile (Q3) = 33–39) for QoL. The items in the QoL-AD reached a median score of 3 except memory, which received a median score of 2. After we grouped the items into Lawton’s four dimensions of QoL the results showed a median score of 3 for all dimensions (Table 2).

**Table 2:** Quality of Life in Alzheimer’s Disease (QoL-AD) scores, rated by persons with dementia (*n* = 164)^b^

Variable	Median	(Q1-Q3)
Total score (range 13-52 ^a^)	36	(33-39)
Items from the QoL-AD questionnaire (range 1-4 ^a^)		
Physical health	3	(2-3)
Energy level	3	(2-3)
Mood	3	(2-3)
Living situation	3	(3-4)
Memory	2	(2-2)
Relations with relatives^c^	3	(3-4)
Relation with wife/husband^d^	3	(3-4)
Relations with friends^e^	3	(3-3)
Self as a whole^f^	3	(2-3)
Chores around the house^e^	3	(2-3)
Things for fun^e^	3	(2-3)
Financial situation^e^	3	(2-3)
Life as a whole^e^	3	(3-3)
Lawton’s dimensions of QoL (range 1-4 ^a^)		
Environmental quality^e^	3	(2-3)
Behavioural competence^e^	3	(2-3)
Perceived QoL^g^	3	(3-3)
Psychological wellbeing^h^	3	(3-3)

^a^ The underlined score is the most favourable score

^b^Missing *n* = 13 (unable to complete QoL-AD due to cognitive problems related to dementia);

^c^missing *n* = 1; ^d^missing *n* = 9; ^e^missing *n* = 2; ^f^missing *n* = 3; ^g^missing *n* = 10; ^h^missing *n* = 4 Q1 = first quartile; Q3 = third quartile; QoL = quality of life

The informal caregiver’s total median CLINT score was 14 (Q1–Q3 = 11–16), indicating overall satisfaction with received health care and social services. Informal caregivers were somewhat more satisfied with staff being honest, food portions and overall health care and services received, than with the other indicators (Table 3).

**Table 3:** Proxy rating of quality of care, by next of kin

Variable	Median	(Q1-Q3)
CLINT score, total (*n* = 150) (range 8–40^a^)	14	(11-16)
Personal interaction (range 1–5^a^)	2	(2-3)
Staff doing what you want them to do (range 1–5^a^)	2	(1-2)
Staff being honest (range 1–5^a^)	1	(1-1)
Hygiene (range 1–5^a^)	2	(1-3)
Cleaning (range 1–5^a^)	2	(1-2)
Food portions (range 1–5^a^)	1	(1-2)
Appreciating meals (range 1–5^a^)	2	(1-2)
Overall satisfaction (range 1–5^a^)	1	(1-2)
	n	(%)
Receiving dementia-specific care	140	(79)
Satisfaction with dementia-specific care		
Very satisfied	83	(60)
Satisfied	49	(35)
Neither satisfied nor dissatisfied	6	(4)
Dissatisfied		
Very dissatisfied	2	(1)
Quality of care indicators (*n* = 177)		
Daily pain^b^	54	(31)
Fall the past month	40	(23)
Injured when falling	16	(40)
Pressure ulcer^c^	1	(0,6)
Weight loss ≥4% the previous year^c^	52	(29)

^a^ The underlined score is the most favourable score

^b^missing *n* = 2; ^c^missing *n* = 15

CLINT = Client Interview instrument

The majority of the sample (79%) received dementia-specific care. Among informal caregiver, 95% were either very satisfied (60%) or satisfied (35%) with received dementia-specific care (Table 3).

Regarding the QoC indicators, 31% of the persons with dementia had daily pain and 29% had suffered a weight loss of ≥4% during the previous year. Furthermore, 23% of the persons with dementia had fallen during the last month and 40% (16/40) of them had sustained injury when falling (Table 3).

Comparing QoL dimensions with the QoC indicators revealed that QoL in the dimensions behavioural competence and psychological wellbeing was significantly lower (z = −2.2, *p* = 0.026, and z = − 2.8, *p* = 0.006, respectively) in persons with dementia expressing signs of daily pain (*n* = 54) compared with those showing no pain (*n* = 121). The results revealed similar differences whether pain less than once a day was included or excluded in the category pain (*p* = 0.029 and *p* = 0.006, respectively). No other significant differences were found between the QoC indicators and the QoL dimensions, or the QoL-AD total score (Table 4).

**Table 4:** Quality of life self-reported by the persons with dementia, and proxy assessment of quality of care

	Behavioral competence			Environmental quality			Perceived Quality of life			Psychological well-being			QoL-AD Total		
	Median	Q1-Q3	*P*-value^1^	Median	Q1-Q3	*P*-value^1^	Median	Q1-Q3	*P*-value^1^	Median	Q1-Q3	*P*-value^1^	Median	Q1-Q3	*P*-value^1^
Pain			.026			.813			.658			.006			.060
Yes	2.3	2.0-2.6		3.0	2.5-3.5		3.0	3.0-3.7		2.7	2.5-3.0		35.0	32.0-39.0	
No	2.6	2.2-2.8		3.0	2.5-3.5		3.0	3.0-3.7		3.0	2.7-3.3		37.0	34.0-39.0	
Fall			.323			.264			.734			.297			.642
Yes	2.2	1.8-2.8		3.0	3.0-3.5		3.0	3.0-4.0		2.7	2.7-3.3		35.0	32.0-40.0	
No	2.6	2.2-2.8		3.0	2.5-3.5		3.0	3.0-3.7		3.0	2.7-3.0		36.0	34.0-39.0	
Injured when fallen			.868			.645			.551			.389			.690
Yes	3.0	2.5-3.5		2.3	2.0-2.8		3.0	3.0-4.0		2.8	2.3-3.6		36.0	32.0-42.0	
No	2.4	1.7-2.9		3.0	3.0-3.5		3.0	3.0-3.8		2.7	2.2-3.3		35.0	31.0-39.0	
Weight loss ≥4%			.729			.273			.542			.636			.591
Yes	2.4	2.0-2.8		3.0	2.5-3.5		3.0	3.0-3.7		3.0	2.7-3.0		36.0	33.0-38.0	
No	2.6	2.0-2.8		3.0	2.5-3.5		3.0	3.0-3.7		2.7	2.3-3.0		36.0	33.0-39.0	
Receiving specific dementia care			.824			.318			.976			.794			.779
Yes	2.5	2.2-2.8		3.0	2.5-3.5		3.0	3.0-3.7		3.0	2.7-3.0		36.0	33.0-39.0	
No	2.6	2.2-2.7		3.0	2.5-3.5		3.0	3.0-3.7		3.0	2.3-3.3		36.0	33.3-39.3	
CLINT Total			.059			**.039**			.067			.220			**.006**
High satisfaction (0-13)	2.6	2.4-2.9		3.0	3.0-3.3		3.0	3.0-3.7		3.0	2.5-3.3		39.0	34.5-40.0	
Low satisfaction (14-40)	2.4	2.2-2.8		3.0	2.5-3.5		3.0	3.0-3.7		2.7	2.7-3.0		35.0	32.3-38.0	

^1^Independent samples Mann-Whitney U-test. Significant values are given in bold

CLINT = Client Interview instrument; QoL-AD = Quality of Life in Alzheimer’s Disease; Q1 = first quartile; Q3 = third quartile

Comparing those with high satisfaction with received health care and social services (CLINT score 0–13, *n* = 60) with those with lower satisfaction (CLINT score 14–40, *n* = 60) showed significantly higher QoL in the dimension environmental quality (z = −2.1, *p* = 0.039) and a significantly higher QoL-AD total score (z = −2.8, *p* = 0.006). However, there were no significant differences in QoL between those receiving dementia-specific care (*n* = 140) and those not receiving dementia-specific care (*n* = 36) (Table 4).

## Discussion

Overall the persons with dementia in this study reported a high total QoL-AD score as well as a high score in the four domains was scored. It should be noted that 68% of the study population co-habited with their informal caregivers which may have affected the results. Previous research found that living alone is significantly associated with lower QoL [8, 40] while a stronger social network contributes to higher QoL [41]. Furthermore, high QoL in persons with dementia living in north and western part of Europe is not an unexpected result. Previous research report that persons aged 65 years or older in the Nordic countries are generally more satisfied with life compared with the average for for their peers in other European countries [42, 43]. Additionally, the informal caregiver reported high satisfaction with health care and social services, according to both the CLINT scores and responses regarding dementia-specific care. The results from this study also reveal that satisfaction with health care and the social services seems to have a positive effect on QoL total scores and the dimension environmental quality. However, this significance was not found for those receiving dementia- specific cares.

Regarding the significance of QoC indicators for QoL, the results reveal that one-third of the persons with dementia in this study had daily pain and that these persons had significantly lower QoL in the dimensions behavioural competence and psychological wellbeing compared with those without daily pain. The dimension behavioural competence contains the individual’s functions and capacity for adaptive behaviour [15] and will probably be further reduced by pain. In the dimension psychological wellbeing, the items mood, self as a whole and life as a whole were negatively affected by pain. We conclude that pain in persons with dementia will probably lead to negative effects such as anxiety, depression, agitation and worrying [15].

Pain has previously been found to be almost doubled in persons with dementia compared with persons without dementia [22]. The difficulties in detecting pain, often communicated via non-verbal behaviour, and presenting as behavioural disorders in persons with dementia, may lead to inadequate treatment with neuroleptics or sedatives rather than analgesic drugs, leading to concealment of pain-related symptoms and consequently hindering tailored treatment of pain [21, 22]. Thus, by identifying and treating underlying causes of pain we may resolve problematic behaviour, relieve pain, and improve QoL in persons with dementia.

Almost one-fourth of the persons with dementia in this study had fallen in the preceding month and a substantial percentage of these (40%) had sustained an injury when falling. However, the results from our study could not detect any effect on QoL regarding these QoC indicators. Earlier research has found that approximately 10% of falls in older people cause injury [44], making the frequency in this study four times higher compared with that for the general population of people aged >65 years. Previous research has also found a significant relationship between falling and dementia [23] and reports that the risk of falling is doubled for persons with dementia compared with older people without cognitive impairment [44]. Moreover, Sweden has been identified as having one of the highest fall-related injury rates (including injuries such as fractures) in the world [44]. The reason is not clear but has been suggested to be associated with heterogeneity in fracture probability and reduced sunlight exposure [44].

The inclusion criterion of being at risk of nursing home admission within six months could be the explanation as to why one-third of persons with dementia in this study had lost ≥4% weight in the preceding year. Weight loss is commonly associated with dementia and seems to increase with the severity and progression of the disease [45]. Persons with dementia develop several feeding difficulties such as changed dietary habits, and physical changes, but also difficulties in preparing food, eating and swallowing [45]. Weight loss is therefore an important predictor for institutionalization [25] and mortality [45], but was not found to have significance for QoL in this study.

### Methodological limitations

The results from this study should be interpreted with caution because of some limitations. Firstly, we report on a specific sample: persons with dementia at risk of nursing home admission. Thus, our results cannot be generalized to all persons with dementia receiving home care. Secondly, the sample may not be representative for the whole of Sweden since the participants were recruited in a selected geographic area and not randomly selected from the national population. Furthermore, home care can differ between different Swedish municipalities since each municipality is independent when it comes to decisions about provision of health care and social services. Consequently, the results may not be representative of all municipalities, thus complicating the generalization of results. On the other hand, the sample was selected from twelve municipalities in both rural and urban areas as well as from both public and private home care organizations.

One possible explanation for the high satisfaction with received health care and social services at home and for the self-reported high QoL could be the 50 drop-outs who either had changed their minds or were too tired to participate. It is possible that they would have rated QoL and QoC lower and that data from their point of view could have affected the results.

Other aspects to consider is that the informal caregivers’ dependency on formal care and services at home and hesitations about negatively evaluating formal care and services. These aspects could have affected the results, which may have led to underreporting of dissatisfaction with care and services. However, to minimize this effect the interviews were carried out independently of the care and services delivered to the persons with dementia.

In dementia research, self-report of QoL is not possible in many cases, as dementia affects cognitive abilities, which raises doubts about the ability of persons with dementia to make valid assessments and give reliable answers regarding their QoL. However, there is a growing body of evidence suggesting that persons with mild to moderate dementia can complete standardized questionnaires on self-reported QoL [37, 46]. The QoL-AD is a self-reported, multi-dimensional instrument specifically designed for persons with Alzheimer’s disease [37]. It has been suggested to be the most widely used self-report QoL instrument internationally because of ease and rapidity of administration (10–15 min) focusing on QoL domains assessed to be important for cognitively impaired older persons [37, 47]. It has been found to be a reliable and valid self-report instrument for persons with Alzheimer’s disease with Mini Mental State Examination (MMSE) scores >10 [37, 48] and is appropriate to use in persons with dementia with MMSE scores as low as 3 [48]. The sample in this study had a median score of 16 on the S-MMSE. Owing to cognitive impairment 13 persons with dementia were unable to answer the QoL-AD questions and nine did not answer the item “relations with wife/husband”, probably because they were either widowed or not married.

It had been possible to make factor analysis then the number of items and the number of participants were enough (10 times more people than items). However, this was judged not to be applicable in this study when the range of variation (IQ) was at most only 2 steps. The second reason was that the sample is specific, i.e. persons with dementia at risk of nursing home admission. Thus, the result of a factor analysis might be difficult to generalize to all persons with dementia receiving home care. However, this could be of interest to further analyze in future studies.

This study used informal caregiver’s perceptions of QoC instead of obtaining responses regarding QoC from the persons with dementia, which would have been a more adequate perspective. However, the difficulties described above using persons with dementia as respondents were the reason for using informal caregiver as proxy raters. It should be noted that proxy ratings may be influenced by the proxy’s own expectations, burden and depression [37] and that this may have affected the results.

Another way of investigating QoC could have been using the interRAI home care quality indicators based on the MDS/RAI [49]. However, the MDS/RAI is not common applied from a Swedish Context since no translation of the form or the manual is yet published.

## Conclusions

In this study, we found a high overall self-reported QoL in persons with dementia and a general satisfaction with received health care and social services at home. With regard to the QoC indicators, only pain was significantly related to lower QoL. However, the results indicate need for improvement of health care and social services since one-third of the persons with dementia had daily pain and had suffered a weight loss of ≥4% during the preceding year. Furthermore, nearly one-fourth had fallen during the preceding month and 40% of these had sustained injury when falling. Registered nurses are responsible for nursing interventions related to pain, patient safety, skin care, prevention of accidents and malnutrition. Therefore, from a nursing perspective, this knowledge about improvable aspects of dementia care is of great importance to enable tailoring of nursing interventions, thereby improving QoC outcomes such as QoL in persons with dementia.

## Abbreviations

ADLs: activities of daily living
CLINT: Client Interview instrument
KATZ-ADL: Katz Index of Independence in Activities of Daily Living
OECD: Organization for Economic Co-operation and Development
Q1: first quartile
Q3: third quartile
QoC: quality of care
QoL: quality of life
QoL-AD: Quality of Life in Alzheimer’s Disease
S-MMSE: Standardized Mini Mental State Examination
